# *catena*-Poly[[dichloridozinc(II)]-μ-1,4-bis­(1*H*-imidazol-1-yl)benzene]

**DOI:** 10.1107/S1600536810044429

**Published:** 2010-11-06

**Authors:** Yi Nan, Ling Yuan, Cheng-Bi Xu, Shan-Ji Nan, Yang Niu

**Affiliations:** a Traditional Chinese Medicine College of Ningxia Medical University, Yinchuan, Ningxia Province 750004, People’s Republic of China; b Pharmacy College of Ningxia Medical University, Yinchuan, Ningxia, Province 750004, People’s Republic of China; c The Second Hospital of Jilin University, Changchun, Jilin Province 130041, People’s Republic of China

## Abstract

In the title one-dimensional coordination polymer, [ZnCl_2_(C_12_H_10_N_4_)]_*n*_, the Zn^II^ atom (site symmetry 2) is coordinated by two chloride ions and two 1,4-bis­(imidazol-1-yl)benzene ligands, generating a distorted tetra­hedral ZnCl_2_N_2_ geometry for the metal ion. The bridging ligand, which is completed by crystallographic inversion symmetry, links the Zn^II^ atoms into zigzag chains propagating in [101]. Within the ligand, the dihedral angle between the central benzene ring and terminal imidazole ring is 27.82 (13)°.

## Related literature

For background to coordination polymers containing imidazole-derived ligands, see: Jin *et al.* (2006 ▶); Li *et al.* (2010 ▶); Lin *et al.* (2008 ▶). 
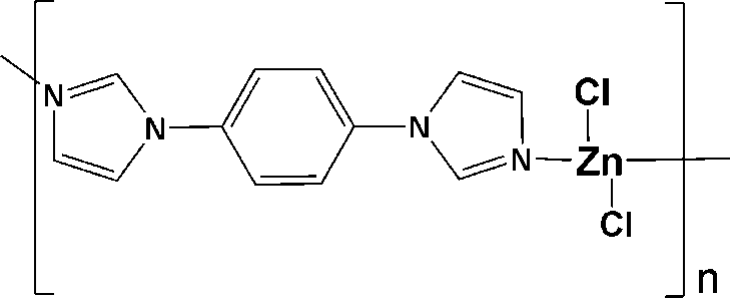


## Experimental

### 

#### Crystal data

[ZnCl_2_(C_12_H_10_N_4_)]
*M* *_r_* = 346.51
Monoclinic, 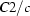

*a* = 13.196 (3) Å
*b* = 6.3780 (13) Å
*c* = 16.431 (3) Å
β = 93.75 (3)°
*V* = 1379.9 (5) Å^3^
*Z* = 4
Mo *K*α radiation
μ = 2.16 mm^−1^
*T* = 293 K
0.25 × 0.22 × 0.20 mm

#### Data collection

Rigaku Mercury area-detector diffractometer
Absorption correction: multi-scan (*CrystalClear*; Rigaku/MSC, 2005 ▶) *T*_min_ = 0.589, *T*_max_ = 0.650
5725 measured reflections
1209 independent reflections
1136 reflections with *I* > 2σ(*I*)
*R*_int_ = 0.028

#### Refinement

*R*[*F* ^2^ > 2σ(*F* ^2^)] = 0.028
*wR*(*F* ^2^) = 0.058
*S* = 1.18
1209 reflections
87 parameters
H-atom parameters constrained
Δρ_max_ = 0.26 e Å^−3^
Δρ_min_ = −0.33 e Å^−3^

### 

Data collection: *CrystalClear* (Rigaku/MSC, 2005 ▶); cell refinement: *CrystalClear*; data reduction: *CrystalClear*; program(s) used to solve structure: *SHELXS97* (Sheldrick, 2008 ▶); program(s) used to refine structure: *SHELXL97* (Sheldrick, 2008 ▶); molecular graphics: *SHELXTL* (Sheldrick, 2008 ▶); software used to prepare material for publication: *SHELXTL*.

## Supplementary Material

Crystal structure: contains datablocks I, global. DOI: 10.1107/S1600536810044429/hb5712sup1.cif

Structure factors: contains datablocks I. DOI: 10.1107/S1600536810044429/hb5712Isup2.hkl

Additional supplementary materials:  crystallographic information; 3D view; checkCIF report

## Figures and Tables

**Table 1:** Selected bond lengths (Å)

Zn1—N1	2.0248 (19)
Zn1—Cl1	2.2643 (8)

**Fig. 1.**
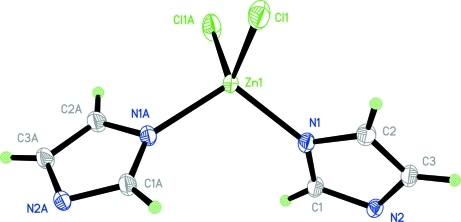
The molecular structure of (I). Displacement ellipsoids are drawn at the 30% probability level and H atoms are shown as small spheres of arbitrary radius.

**Fig. 2.**
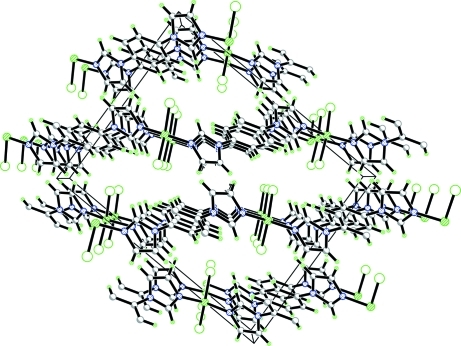
The crystal packing for (I).

## supplementary crystallographic information

### Comment

Imidazole derivates has been well used in crystal engineering, and a large number of imidazole-containing flexible ligands have been extensively studied (Jin *et al.*, 2006; Lin *et al.*, 2008). However, to our knowledge, the research on imidazole ligands bearing rigid spacers is still less developed (Li *et al.*, 2010).

Single-crystal X-ray diffraction analysis reveals that the title compound (I) crystallizes in the monoclinic space group *C2/c*. The geometry of the Zn(II) ion is surrounded by two imidazole rings of distinct *L* ligands and two chlorine anions, which illustrates a slightly distorted tetrahedral coordination environment (Fig 1). Notably, as shown in Fig 2, the four-coordinated Zn(II) center is connected by the linear ligand *L* into an infinite one-dimensional zigzag chain.

### Experimental

A mixture of C_2_H_5_OH and H_2_O (1:1, 8 ml), as a buffer layer, was carefully layered over a solution of ZnCl_2_ (0.02 mmol) in H_2_O (6 ml). Then a solution of 1,4-Bis(imidazol-1-yl)phenyl (L, 0.06 mmol) in C_2_H_5_OH (6 ml) was layered over the buffer layer, and the resultant reaction was left to stand at room temperature. After *ca* three weeks, colorless blocks of (I) appeared at the boundary. Yield: ~30% (based on L).

### Refinement

C-bound H atoms were positioned geometrically and refined in the riding-model approximation, with C—H = 0.93 Å and *U*_iso_(H) = 1.2 *U*_eq_.

### Crystal data

**Table:**

[ZnCl_2_(C_12_H_10_N_4_)]	*F*(000) = 696
*M**_r_* = 346.51	*D*_x_ = 1.668 Mg m^−^^3^
Monoclinic, *C*2/*c*	Mo *K*α radiation, λ = 0.71073 Å
Hall symbol: -C 2yc	Cell parameters from 6568 reflections
*a* = 13.196 (3) Å	θ = 6.2–54.8°
*b* = 6.3780 (13) Å	µ = 2.16 mm^−^^1^
*c* = 16.431 (3) Å	*T* = 293 K
β = 93.75 (3)°	Block, colorless
*V* = 1379.9 (5) Å^3^	0.25 × 0.22 × 0.20 mm
*Z* = 4	

### Data collection

**Table:**

Rigaku Mercury diffractometer	1209 independent reflections
Radiation source: fine-focus sealed tube	1136 reflections with *I* > 2σ(*I*)
graphite	*R*_int_ = 0.028
Detector resolution: 9 pixels mm^-1^	θ_max_ = 25.0°, θ_min_ = 3.1°
ω scans	*h* = −15→15
Absorption correction: multi-scan (*CrystalClear*; Rigaku/MSC, 2005)	*k* = −7→7
*T*_min_ = 0.589, *T*_max_ = 0.650	*l* = −19→19
5725 measured reflections	

### Refinement

**Table:**

Refinement on *F*^2^	Primary atom site location: structure-invariant direct methods
Least-squares matrix: full	Secondary atom site location: difference Fourier map
*R*[*F*^2^ > 2σ(*F*^2^)] = 0.028	Hydrogen site location: inferred from neighbouring sites
*wR*(*F*^2^) = 0.058	H-atom parameters constrained
*S* = 1.18	*w* = 1/[σ^2^(*F*_o_^2^) + (0.022*P*)^2^ + 1.5504*P*] where *P* = (*F*_o_^2^ + 2*F*_c_^2^)/3
1209 reflections	(Δ/σ)_max_ < 0.001
87 parameters	Δρ_max_ = 0.26 e Å^−^^3^
0 restraints	Δρ_min_ = −0.33 e Å^−^^3^

### Special details

**Table:**

Geometry. All e.s.d.'s (except the e.s.d. in the dihedral angle between two l.s. planes) are estimated using the full covariance matrix. The cell e.s.d.'s are taken into account individually in the estimation of e.s.d.'s in distances, angles and torsion angles; correlations between e.s.d.'s in cell parameters are only used when they are defined by crystal symmetry. An approximate (isotropic) treatment of cell e.s.d.'s is used for estimating e.s.d.'s involving l.s. planes.
Refinement. Refinement of *F*^2^ against ALL reflections. The weighted *R*-factor *wR* and goodness of fit *S* are based on *F*^2^, conventional *R*-factors *R* are based on *F*, with *F* set to zero for negative *F*^2^. The threshold expression of *F*^2^ > σ(*F*^2^) is used only for calculating *R*-factors(gt) *etc*. and is not relevant to the choice of reflections for refinement. *R*-factors based on *F*^2^ are statistically about twice as large as those based on *F*, and *R*- factors based on ALL data will be even larger.

### Fractional atomic coordinates and isotropic or equivalent isotropic displacement parameters (Å^2^)

**Table:**

	*x*	*y*	*z*	*U*_iso_*/*U*_eq_	
Zn1	0.5000	0.47523 (6)	0.2500	0.02629 (14)	
Cl1	0.62428 (6)	0.67534 (12)	0.20142 (4)	0.0488 (2)	
N1	0.45231 (15)	0.2933 (3)	0.15401 (11)	0.0288 (5)	
N2	0.37084 (14)	0.0812 (3)	0.06614 (11)	0.0275 (5)	
C1	0.38454 (18)	0.1419 (4)	0.14535 (14)	0.0296 (6)	
H1A	0.3506	0.0842	0.1878	0.036*	
C2	0.4850 (2)	0.3299 (4)	0.07706 (15)	0.0363 (6)	
H2A	0.5341	0.4274	0.0648	0.044*	
C3	0.4350 (2)	0.2028 (4)	0.02255 (15)	0.0362 (6)	
H3A	0.4422	0.1978	−0.0333	0.043*	
C4	0.30781 (18)	−0.0863 (4)	0.03321 (14)	0.0269 (5)	
C5	0.2728 (2)	−0.0807 (4)	−0.04871 (15)	0.0350 (6)	
H5A	0.2882	0.0330	−0.0811	0.042*	
C6	0.28494 (19)	−0.2555 (4)	0.08161 (15)	0.0334 (6)	
H6A	0.3084	−0.2588	0.1362	0.040*	

### Atomic displacement parameters (Å^2^)

**Table:**

	*U*^11^	*U*^22^	*U*^33^	*U*^12^	*U*^13^	*U*^23^
Zn1	0.0337 (2)	0.0263 (2)	0.0182 (2)	0.000	−0.00328 (15)	0.000
Cl1	0.0571 (5)	0.0594 (5)	0.0295 (4)	−0.0279 (4)	0.0008 (3)	−0.0005 (3)
N1	0.0349 (12)	0.0296 (11)	0.0214 (11)	−0.0060 (9)	−0.0017 (8)	−0.0011 (8)
N2	0.0336 (11)	0.0267 (11)	0.0216 (10)	−0.0062 (9)	−0.0020 (8)	−0.0024 (8)
C1	0.0340 (13)	0.0334 (14)	0.0214 (13)	−0.0070 (11)	0.0014 (10)	−0.0014 (10)
C2	0.0478 (16)	0.0338 (15)	0.0276 (14)	−0.0150 (12)	0.0031 (11)	−0.0001 (11)
C3	0.0529 (17)	0.0344 (14)	0.0213 (13)	−0.0146 (13)	0.0032 (11)	−0.0013 (10)
C4	0.0299 (13)	0.0270 (12)	0.0235 (12)	−0.0032 (10)	−0.0016 (10)	−0.0034 (10)
C5	0.0477 (16)	0.0306 (14)	0.0258 (13)	−0.0086 (12)	−0.0033 (11)	0.0055 (10)
C6	0.0431 (15)	0.0351 (14)	0.0205 (12)	−0.0067 (12)	−0.0080 (10)	0.0004 (10)

### Geometric parameters (Å, °)

**Table:**

Zn1—N1	2.0248 (19)	C2—C3	1.348 (3)
Zn1—N1^i^	2.0248 (19)	C2—H2A	0.9300
Zn1—Cl1^i^	2.2643 (8)	C3—H3A	0.9300
Zn1—Cl1	2.2643 (8)	C4—C6	1.385 (3)
N1—C1	1.317 (3)	C4—C5	1.395 (3)
N1—C2	1.382 (3)	C5—C6^ii^	1.382 (3)
N2—C1	1.359 (3)	C5—H5A	0.9300
N2—C3	1.382 (3)	C6—C5^ii^	1.382 (3)
N2—C4	1.438 (3)	C6—H6A	0.9300
C1—H1A	0.9300		
			
N1—Zn1—N1^i^	110.08 (11)	C3—C2—N1	109.7 (2)
N1—Zn1—Cl1^i^	113.71 (6)	C3—C2—H2A	125.1
N1^i^—Zn1—Cl1^i^	104.11 (6)	N1—C2—H2A	125.1
N1—Zn1—Cl1	104.11 (6)	C2—C3—N2	106.4 (2)
N1^i^—Zn1—Cl1	113.71 (6)	C2—C3—H3A	126.8
Cl1^i^—Zn1—Cl1	111.38 (5)	N2—C3—H3A	126.8
C1—N1—C2	105.98 (19)	C6—C4—C5	120.2 (2)
C1—N1—Zn1	132.71 (16)	C6—C4—N2	120.3 (2)
C2—N1—Zn1	121.06 (16)	C5—C4—N2	119.4 (2)
C1—N2—C3	106.79 (19)	C6^ii^—C5—C4	119.8 (2)
C1—N2—C4	127.6 (2)	C6^ii^—C5—H5A	120.1
C3—N2—C4	125.50 (19)	C4—C5—H5A	120.1
N1—C1—N2	111.1 (2)	C5^ii^—C6—C4	120.0 (2)
N1—C1—H1A	124.5	C5^ii^—C6—H6A	120.0
N2—C1—H1A	124.5	C4—C6—H6A	120.0
			
N1^i^—Zn1—N1—C1	55.1 (2)	N1—C2—C3—N2	−1.0 (3)
Cl1^i^—Zn1—N1—C1	−61.2 (2)	C1—N2—C3—C2	0.5 (3)
Cl1—Zn1—N1—C1	177.4 (2)	C4—N2—C3—C2	−175.6 (2)
N1^i^—Zn1—N1—C2	−131.4 (2)	C1—N2—C4—C6	−25.8 (4)
Cl1^i^—Zn1—N1—C2	112.23 (19)	C3—N2—C4—C6	149.4 (3)
Cl1—Zn1—N1—C2	−9.2 (2)	C1—N2—C4—C5	156.4 (2)
C2—N1—C1—N2	−0.9 (3)	C3—N2—C4—C5	−28.4 (4)
Zn1—N1—C1—N2	173.23 (16)	C6—C4—C5—C6^ii^	0.0 (4)
C3—N2—C1—N1	0.3 (3)	N2—C4—C5—C6^ii^	177.8 (2)
C4—N2—C1—N1	176.3 (2)	C5—C4—C6—C5^ii^	0.0 (4)
C1—N1—C2—C3	1.2 (3)	N2—C4—C6—C5^ii^	−177.8 (2)
Zn1—N1—C2—C3	−173.77 (17)		

Symmetry codes: (i) −*x*+1, *y*, −*z*+1/2; (ii) −*x*+1/2, −*y*−1/2, −*z*.
